# “They Are Not Hard-to-Reach Clients. We Have Just Got Hard-to-Reach Services.” Staff Views of Digital Health Tools in Specialist Mental Health Services.

**DOI:** 10.3389/fpsyt.2019.00344

**Published:** 2019-05-10

**Authors:** Sandra Bucci, Natalie Berry, Rohan Morris, Katherine Berry, Gillian Haddock, Shôn Lewis, Dawn Edge

**Affiliations:** ^1^Division of Psychology and Mental Health, School of Health Sciences, Manchester Academic Health Science Centre, University of Manchester, Manchester, United Kingdom; ^2^Greater Manchester Mental Health NHS Foundation Trust, Manchester, United Kingdom

**Keywords:** psychosis, digital health, mHealth, qualitative, mental health, app

## Abstract

**Background:** Digital health products designed to help people with severe mental health problems appear to be feasible, acceptable, and efficacious. The challenge facing the digital mental health field is implementing digital tools in routine service delivery. To date, there has been a paucity of qualitative research exploring staff views of digital health solutions in the context of mental healthcare. Engaging and involving frontline staff in the design and rollout of new technology to improve utilization is imperative for successful uptake and adoption of digital tools. The aim of the current study is to explore frontline staff views regarding the utility and appropriateness of using digital tools in the healthcare pathway for people accessing specialist secondary care mental health services.

**Method:** Qualitative study using framework analysis was used with 48 mental health staff working in early intervention for psychosis services. Six groups comprising 5–10 early intervention service staff members in each group were conducted across the Northwest of England. Robust measures were used to develop a stable framework, including member checking, triangulation, and consensus meetings.

**Results:** Three themes were identified *a priori*: i) perceived barriers to adopting smartphone apps for early psychosis; ii) acceptability of digital health tools for early psychosis patients; and iii) data security, safety, and risk. Alongside exploring the *a priori* topics, one theme was generated *a posteriori*: iv) relationships.

**Conclusions:** Staff working in specialist early intervention for psychosis services found digital tools on the whole acceptable in mental health service provision, but raised a number of concerns that will likely affect implementation of such systems into routine service delivery and practice. Thirteen recommendations are made in this paper as a result of the themes generated in these data. Implementing of digital systems needs to be simple and uncomplicated and improve clinical workflows for staff rather than hinder and increase clinical workflows. Furthermore, organizational support with a clear plan for implementing technological innovations is required for successful adoption of digital systems. Consideration of staff views around digital systems is important if successful adoption and implementation of such systems are to occur.

Clinical Trial Registration: http://www.isrctn.com, identifier ISRCTN34966555.

## Introduction

Psychosis is associated with high individual, societal, and financial costs and is a public health challenge (1). Life expectancy is reduced by 17–20 years compared to the general population, noted in the Five-Year Forward View as one of the greatest health inequalities in England (2). Treatment for psychosis is time-sensitive, with delays resulting in unplanned inpatient care for relapse. Unscheduled episodes of acute care for management of relapse continue to be major cost drivers for services. The challenge is to improve outcomes through personalized care. Digital health interventions (DHIs) can be used to deliver ecologically valid interventions at the point of need (3). Several digital tools have been developed to assist people experiencing psychosis, with promising findings (4–7). A few studies have explored clinicians’ views of various digital mental health platforms, including computerized and mobile tools (1, 8–11), but these findings have generally been limited to mental health problems more generally, rather than psychosis specifically.

Early psychosis is ta critical period of development (12), and specialist early intervention for psychosis services (EIS) have been designed to offer an intensive model of care in order to minimize the impact of potential relapse. Digitizing health services is a UK National Health Service (NHS) policy priority (13), and technological innovations and solutions are being considered in an attempt to address the size and scale of mental health problems worldwide (14). However, implementation has been challenging, chiefly due to lack of staff engagement (15). Drawing on lessons learned from integrating digital tools within other large institutions, Lluch (16) identified factors including organizational structures, perceived depersonalization of healthcare, and lack of incentives for clinicians as major barriers to implementation. Engaging and involving frontline staff in the design and rollout of new technology to improve utilization is therefore imperative. Furthermore, understanding implementation facilitators and barriers from frontline staff perspectives is important as staff views and beliefs about the utility of digital tools in mental healthcare delivery will undoubtedly influence uptake and the possibility of embedding digital systems and tools in services (17).

Currently, clinicians’ perceptions of integrating digital tools into specialist psychiatric services are unknown. Without such insights, we run the risk of failing to engage staff in coproducing solutions and successfully implementing potentially transformative care. The aim of the current investigation is to explore clinicians’ views regarding the utility and appropriateness of using digital tools in the healthcare pathway for people receiving treatment from early intervention for psychosis services (EIS) in the Northwest of England, UK.

## Materials and Methods

This was a qualitative study nested within a broader research program on the development and testing of a theory-informed smartphone system for early psychosis, Actissist (4). Throughout the life of the Actissist project, we consulted with an expert reference group (ERG) comprising service users, clinicians, and computer scientists. The ERG met regularly to feed into all phases of the project, helping to inform the design of the app and assisting with the analysis and interpretation of study data. Data were gathered from 48 staff working in EIS services. Ethical approval was granted by the National Research Ethics Committee West Midlands–South Birmingham (14/WM/0118). We used a systematic, nonprobabilistic sampling approach to recruit a maximum-variation purposive sample of staff from various roles and disciplines working in EIS. The study was collaborative, involving clinicians in study design, topic guide development, and checking of data themes. Our aims were to understand staff views around digital technology in mental healthcare and to produce findings with some (albeit cautious) transferability beyond our specific study and service setting. While recognizing the inherent subjectivity of this work, such as acknowledgment of our own roles as researchers and clinicians in the research process, we nonetheless assume a critical realist epistemological position. Critical realism combines ontological realism (the world is understood as having a concrete reality outside of human constructions of it) with epistemological relativity.

### Data Collection

A researcher contacted team managers about the study and sought permission to hold a series of focus groups within clinicians’ working day. For three of the six focus groups conducted, the team manager advertised the study during team meetings. Clinicians who expressed an interest to take part attended an estimated 90-min focus group that was scheduled at a time that was convenient for most responders. The remaining three groups were carried out with all staff members of a local early intervention team (split into three groups) as part of the team’s annual “away” day. All participants were aware that the aim of the focus group was to gather staff views of DHIs in the context of mental healthcare. More specifically, staff were informed that the aim of the focus group was to gather healthcare professional views around how to best develop an app for early psychosis service users, ways in which we can engage service users to use a self-management app, incentives and barriers to DHI use in the mental health service setting, and any other general thoughts, both positive and negative, about service users using technology to manage their mental health problems. Staff participants were also aware that the research team would be recruiting participants for a DHI trial in early psychosis following these focus groups.

Eligibility criteria were: ability to provide informed consent, employed staff member of an EIS service in the Northwest of England, English speaking, willing to consent to group being digitally recorded, and consent to publication of deidentified data. Consenting participants were interviewed in focus groups at the team base using a flexible, semistructured topic guide (available on request) developed for the study based on a review of the literature and informed by Smith’s (18) guidelines and our ERG. The topic guide broadly explored staff perceptions of the acceptability of digital health tools; incentives and barriers to the use and implementation of digital health systems; disclosure of risk information; and concerns about data privacy, surveillance, and confidentiality. Designed to reflect naturalistic conversations about specific topics, focus groups are less susceptible to researcher bias than one-to-one researcher-led interviews as participants’ views and group dynamics ultimately shape the data generated (19). However, without skilled facilitation, this can silence some participants (20). The focus groups, averaging 87 min duration, were conducted by experienced facilitators SB, RM, and KB as part of an iterative and inductive process of data collection and analysis. Data collection ceased when no further themes were advanced (i.e., data saturation) (21). The order in which topics emerged was influenced by the topic guide but was not exclusively driven by it. Interviews were digitally recorded and transcribed verbatim.

### Data Analysis

Data were analyzed using a framework analysis approach (22). Although framework analysis shares common features with other qualitative approaches such as thematic analysis, framework methodology makes explicit a visible, systematic process that allows for inclusion of both *a priori* and emergent themes. In collaboration with our ERG, SB, RM, DE, and NB developed the initial framework reflecting important areas we wished to seek views about before further developing the framework. The topic guide essentially informed the framework’s *a priori* themes. After independently coding several transcripts, we refined the framework and subsequent iterations in consultation with the wider team, comprising academic researchers and secondary care clinicians, to discuss and refine the analytic process. Development of the framework involved the nonlinear key stages described in **Table 1**. Codes were organized using NVivo (version 10) software. Regular consensus meetings were held until a stable framework emerged. The analytical framework was not complete until all transcripts were coded and quality assurance measures completed, including independent peer verification of the framework, triangulation of analysis, and member checking of the analytical matrix (22).

**Table 1:** Description of the analytic process.

Stage of analysis	Description
1. Familiarization with the data	Listening to recordings, reading and rereading transcripts, making analytical notes.
2. Coding the data	Includes both deductive (using predefined codes based on specific research questions) and inductive approaches (using “open coding” to identify any emergent, possibly relevant information).
3. Developing the thematic framework	Initial framework developed through comparing codes assigned to the data after independently coding several transcripts and agreeing on the set of codes to be assigned to subsequent transcripts. Data were interpreted and summarized, new codes generated, redundant codes deleted, and overlapping codes merged.
4. Indexing	The framework was applied to the data set.
5. Charting	Charting a framework matrix for each emergent category across the whole data set was developed using QSR International’s NVivo 10 data management software.
6. Mapping and interpretation	Emergent (*a posteriori*) and *a priori* characteristics of the data were identified and connections between categories mapped, facilitating exploration of relationships (similarities and differences) and theoretical concepts and generation of typologies.

### Reflexivity

Reflexivity refers to the process of acknowledging the team’s subjective experiences and how this may influence the process of the analysis process (23). SB, KB, and GH are qualified clinical psychologists who have worked extensively with individuals with psychosis as well as services/clinicians involved in delivering mental healthcare in the public health service. At the time of analysis, RM and NB were experienced research assistants working with people with psychosis in the context of research trials. SL is an adult psychiatrist with many years’ experience working with people with severe mental health problems. DE is an experienced qualitative researcher. We acknowledge that our shared knowledge and experiences may have had an impact upon interpretation of the data.

## Results

The sample, broken down by group in **Table 2**, consisted of six groups comprising 5–10 EIS staff members in each group (*N* = 48 participants). Participants were mainly white British (*n* = 40), with a total mean age across focus groups of 31.6 (range: 19–50) and from a range of professions, including care coordinators (*n* = 10); clinical psychologists (*n* = 8); mental health practitioners (*n* = 5); team managers (*n* = 5); support, time, and recovery (STR) workers (*n* = 5); community psychiatric nurses (*n* = 4); social workers (*n* = 4); psychiatrists (*n* = 4); researchers (*n* = 2); and a team secretary (*n* = 1). Years of professional experience working with EIS service users ranged from 4 months to 22 years. The majority of staff used a smartphone themselves (*n* = 42), and of these, 38 used apps in their personal life. Many staff also stated that they used email and text messages (*n* = 36) to liaise with service users. All teams approached agreed to participate in the study.

**Table 2:** Participant demographics and technology use by focus group.

	Focus group 1 (*n* = 6)	Focus group 2 (*n* = 9)	Focus group 3 (*n* = 10)	Focus group 4 (*n* = 9)	Focus group 5 (*n* = 5)	Focus group 6 (*n* = 9)	Total (*n* = 48)
**Mean age (range)**	38 (30–50)	33.6 (28–40)	34 (30–41)	36 (32–50)	35.2 (27–49)	36.1 (19–43)	36.2 (19–50)
**Gender**	Female: *n* = 4 Male: *n* = 2	Female: *n* = 5 Male: *n* = 4	Female: *n* = 6 Male: *n* = 3 Missing: *n* = 1	Female: *n* = 4 Male: *n* = 5	Female: *n* = 4 Male: *n* = 1	Female: 4 Male: 5	Female: 27 Male: 20 Missing: 1
**Ethnicity**	White British: *n* = 4 Mixed: *n* = 1 White Irish: *n* = 1	White British: *n* = 9	White British: *n* = 7 Mixed: *n* = 2 Missing: *n* = 1	White British: *n* = 7 Mixed: *n* = 1 Missing: *n* = 1	White British: *n* = 5	White British: *n* = 8 Missing: *n* = 1	White British: *n* = 40 Mixed: *n* = 4 White Irish: *n *= 1 Missing: *n* = 3
**Job title**	Clinical psychologist: *n* = 3 Researcher: *n* = 2 Team manager: *n* = 1	Care coordinator: *n* = 3 Community Psychiatric Nurse (CPN): *n* = 1 Psychiatrist: *n* = 1 Social worker: *n* = 2 STR worker: *n* = 1 Team manager: *n* = 1	Care coordinator: *n* = 4 Clinical psychologist: *n* = 1 Assistant clinical psychologist: *n* = 1 Consultant psychiatrist: *n* = 1 CPN: *n* = 1 Mental health practitioner: *n* = 1 Team manager: *n* = 1	Care coordinator: *n* = 3 CPN: *n* = 1 Psychiatrist: *n* = 1 Social worker: *n* = 2 STR worker: *n* = 1 Team manager: *n* = 1	Consultant clinical psychologist: *n* = 1 Trainee clinical psychologist: *n* = 1 Mental health practitioner: *n *= 2 Psychiatrist: *n* = 1	Clinical psychologist: *n* = 1 Mental health practitioner: *n* = 2 Student mental health nurse: *n* = 1 STR worker: *n* = 3 Team manager: *n* = 1 Team secretary: *n* = 1	Care coordinator: *n* = 10 Clinical psychologist: *n* = 8 CPN: *n* = 4 Mental health practitioner: *n* = 5 Psychiatrist: *n* = 4 Researcher: *n* = 2 Social worker: *n* = 4 STR worker: *n* = 5 Team manager: *n* = 5 Team secretary: *n* = 1
**Mean time in post**	3 years 3 months	2 years 1 month	4 years 1 month	3 years	3 years 2 months	7 years 4 months	
**Mean time working with EIS service users**	10 years 1 month	2 years 3 months	4 years 4 months	6 years 6 months	4 years 1 month	5 years 3 months	
**Smartphone ownership**	Yes: *n* = 5 No: *n* = 1	Yes: *n* = 7 No: *n* = 2	Yes: *n* = 10	Yes: *n* = 8 No: *n* = 1	Yes: *n* = 4 No: *n* = 1	Yes: *n* = 8 No: *n* = 1	Yes: *n* = 42 No: *n* = 6
**Smartphone app use**	Yes: *n* = 5 No: *n* = 1	Yes: *n* = 6 No: *n* = 2 Missing: *n* = 1	Yes: *n *= 8 No: *n* = 2	Yes: *n* = 7 No: *n* = 2	Yes: *n* = 4 No: *n* = 1	Yes: *n* = 8 No: *n* = 1	Yes: *n* = 38 No: *n* = 9 Missing: *n* = 1
**Contact with service users *via* email or texts**	Yes: *n* = 5 No: *n* = 1	Yes: *n* = 7 No: *n* = 2	Yes: *n* = 9 No: *n* = 1	Yes: *n* = 7 No: *n* = 2	Yes: *n* = 2 No: *n* = 2 Missing: *n* = 1	Yes: *n* = 6 No: *n* = 2 Missing: *n *= 1	Yes: *n* = 36 No: *n* = 10 Missing: *n* = 2
**Contact with service users *via* email or texts using a smartphone**	Yes: *n* = 1 No: *n* = 5	Yes: *n* = 1 No: *n* = 8	Yes: *n* = 2 No: *n* = 8	Yes: *n* = 2 No: *n* = 7	Yes: *n* = 1 No: *n* = 4	Yes: *n* = 1 No: *n* = 7 Missing: *n* = 1	Yes: *n* = 8 No: *n* = 39 Missing: *n* = 1

STR, support, time, and recovery; EIS, early intervention for psychosis services.

**Figure 1:**
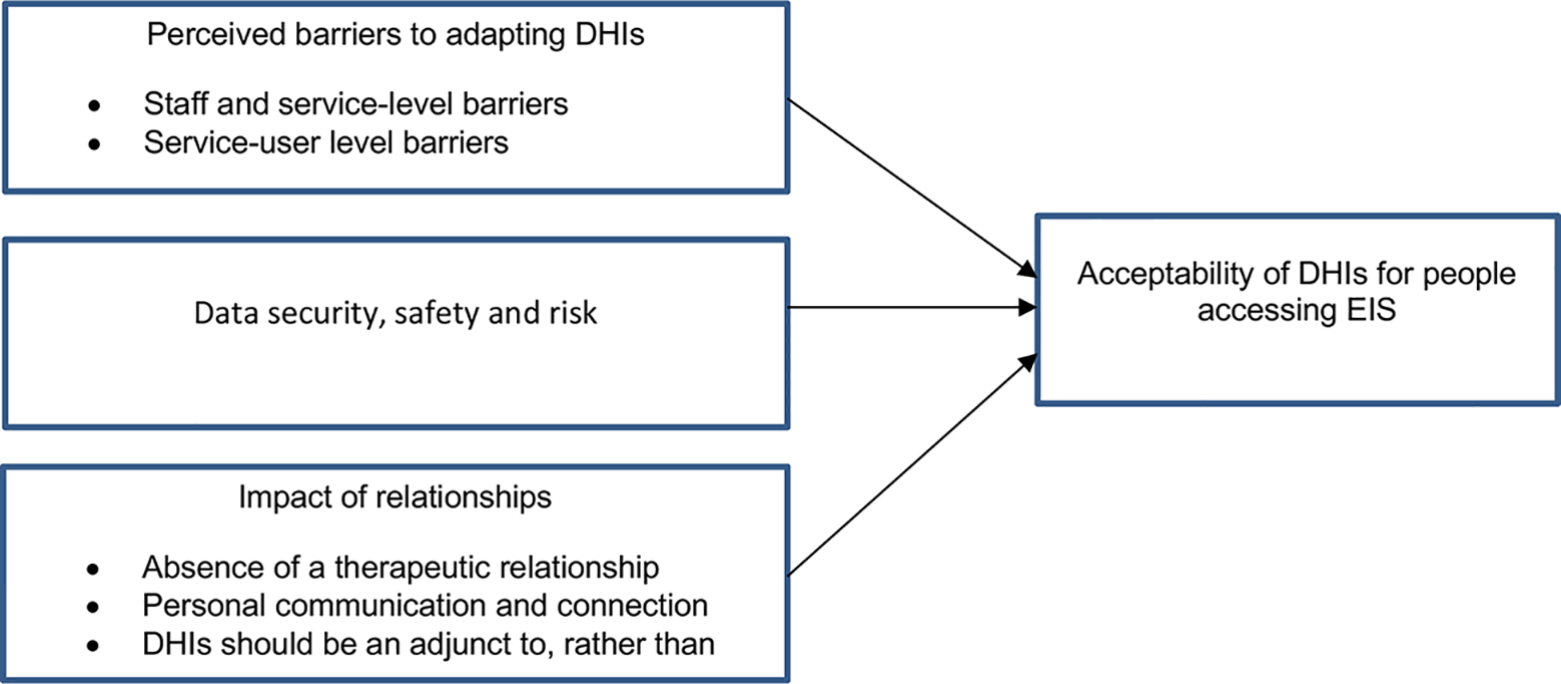
Framework structure of themes.

Three themes were established *a priori*: i) perceived barriers to adopting smartphone apps for early psychosis; ii) acceptability of digital health tools for early psychosis patients; and iii) data security, safety, and risk. Alongside exploring the *a priori* topics, one theme was generated *a posteriori*: iv) relationships. An illustrative diagram of the framework is presented in **Figure 1** and described and elaborated below, evidenced by anonymized quotes embedded within the text.

### Theme 1: Perceived Barriers to Adopting Digital Health Interventions

Although staff described several potential benefits of DHIs for early psychosis, perceived barriers were also noted and are separated here by staff and service–level barriers and service user–level barriers.

#### Staff and Service–Level Barriers

Staff felt that resources would be better spent on investing in staff training rather than developing technology that could ultimately replace clinician skills, as well as deskill and threaten the workforce. As such, the perceived threat of apps usurping the clinician’s role may be a barrier to staff recommending digital health approaches to healthcare:

*Resources are channeled into technological advancement when perhaps it would be better channeled into staffing and training* (Participant 9, Group 2).

Staff detailed concerns regarding their own ability to use technology and expressed doubt around their own skills, familiarity, and confidence in using technology. This highlighted the need for resources to be put toward not only service users but also staff to ensure they are fully trained and offered support in the use of DHIs:

*There’s no point in [a patient] being a whizz on that computer and smartphone and me not having a clue cos I wouldn’t be able to support adequately, there would be … ongoing training needs* (Participant 35, Group 5).

Staff members identified a disparity between their own use of technology and the younger generation’s adoption of technology in their day-to-day life, recognizing that digital tools are culturally relevant for the younger so-called digital native generation who communicate and interact regularly with others using digital technology. For example, some of the older members of the focus groups referred to themselves or others in the service as “old-fashioned” or “old-school,” reflecting some reluctance to move away from more traditional, face-to-face pathways of care:

*I think sometimes people are, for whatever reason, don’t think that’s a good way of communicating; they like the old-fashioned way* (Participant 33, Group 4).

However, some staff also noted that despite not feeling entirely confident with technology themselves due to age and experience, they were willing to adapt to incorporate technology within their own clinical practice for patient need:

*I consider myself a bit of a dinosaur with things like technology, but it doesn’t stop me using certain things … I use Skype to contact one of my clients who just wouldn’t leave the room; I couldn’t access her any other way, so I’m not opposed to the idea of using technology* (Participant 26, Group 4).

Staff members also described low expectations and confidence in the ability of the NHS to implement digital tools within mental health services. Such perceptions stemmed from past negative experiences of the NHS having a “poor reputation of large-scale IT projects and even delivering small-scale IT projects with any level of competence” (Participant 31, Group 4). As such, some staff expressed clear mistrust of NHS technology adoption and preferred paper-and-pencil methods of healthcare monitoring:

*There’s something about trying to change with the times, but I also quite like to write something down on a bit of paper, and you know it’s not going to break down or you’re not going to lose your internet connection* (Participant 39, Group 5).

Additionally, the level of burden placed on clinicians regarding time constraints and the potentially complicated process of coping with real-time data workflows that digital platforms can produce, as well as managing self-monitoring data input by service users themselves, was perceived to be a potential barrier that would need to be addressed to improve the likelihood of successful service implementation. Staff described the need for digital tools and web servers holding the data to be easily accessible, uncomplicated, and able to complement, rather than hinder, clinical practice:

*If it is going to be another ten passwords, or you go in a special room to access that device to look at all other things, then you are actually thinking in the real day of a care coordinator’s life where there’s already so much administration that a new thing also had to be easy to use and seen as a worthwhile tool* (Participant 29, Group 4).

#### Service User–Level Barriers

The most prevalent service user–related barrier, as perceived by staff, related to the “digital divide.” Staff felt that some service users would not own or be able to afford to use smartphone technology, rendering them unable to access digital healthcare solutions. Additionally, some staff reported that a large proportion of EIS service users did not speak English as their first language or had poor literacy skills, including digital literacy skills, and would, therefore, find it difficult to understand the information presented in digital tools such as apps. This concept of the digital divide led to perceived concerns that apps would only “be for the benefit of a real minority of people” (Participant 9, Group 2) and disadvantage people from low socioeconomic backgrounds in particular:

*[For] people on benefits, phones are considered a luxury item … and if people aren’t working because of health needs, essentially they are being denied healthcare because they can’t afford a phone* (Participant 31, Group 4).

Concerns were also expressed about the potential impact of symptoms on service users’ abilities and willingness to engage with digital technology. For example, many staff provided examples of working with service users who had symptoms associated with psychosis that were directly related to technology or expressed concerns that a smartphone app may exacerbate symptoms:

*One of my clients would have a really big issue, he wouldn’t like it cos all his delusional beliefs are about the Internet and computer symptoms…* (Participant 22, Group 3).

*I think with a lot of clients, the symptoms can be similar … in terms of them feeling monitored or watched, and that could add to the distress if you say, “Here’s an app, we’re going to monitor what you’re doing”* (Participant 27, Group 4).

Clinicians also raised concerns that low levels of motivation would prevent some service users from fully engaging with digital healthcare approaches. There was the feeling that apps specifically should use schedules and prompts to remind people to use the device and use should be incentivized, avoid repetition, and include a social media component (if possible) to facilitate connection with others and enhance engagement with the device:

*A mobile phone app is never going to be able to engage people and motivate people, but there may be a way to do it … you offer some way of engaging them, like there’s a progression, like you know our avatar grows or you get to a certain level you get and you get credit for your phone* (Participant 28, Group 4).

### Theme 2: Acceptability of Digital Health Interventions for People Accessing Early Intervention for Psychosis Services

Staff viewed digital technology as a generally acceptable approach to healthcare and highlighted a number of potential benefits. One such perceived benefit was that apps are easily accessible in any place, at any time. In this sense, smartphone apps more specifically were seen to have wider reach in comparison to computerized interventions.

*You don’t have to wait till you get home for your computer … it is [available] there and then* (Participant 3, Group 1).

The on-demand access inherently available within an app was also viewed as being able to provide more ecologically valid assessment of symptoms [“The person would be recording their experiences immediately … in a way where you don’t necessarily get to access when you are seeing someone on a weekly basis” (Participant 45, Group 6)]. Furthermore, staff acknowledged that digital tools could extend the reach of services to people who may struggle to access traditional services:

*So generally speaking, they are not hard-to-reach clients. We have just got hard-to-reach services, but we know that it is tough for some people to get to services…* (Participant 2, Group 1).

Participants viewed apps as potentially destigmatizing due to the now-everyday nature of a mobile phone and availability of an app, which could normalize both the experience of psychosis and the act of self-managing one’s health:

Participant 4: *It is potentially really non-stigmatizing in that people can access it as long as the app is very, very discrete … it just looks like you’re texting or just using a normal app or something*.

Participant 2: *Yeah. I wonder if the fact that there is an app for it, if that makes it more normalizing in itself* (Group 1).

The majority of staff felt that smartphones are contemporary, progressive, modern, and relevant. Using digital technology in the context of healthcare can reflect the way in which individuals currently communicate with one another. In this sense, the use of digital tools such as apps to help manage symptoms and well-being was viewed as an acceptable method as “it fits with the modern day” (Participant 6, Group 1).

Staff also felt that digital tools have the potential for people to feel more empowered in their healthcare, affording them more control and choice over the way they engage with and receive mental healthcare, as well as the location and time of accessing support:

*It could be a bit more empowering for the service user not to be reliant on a member of staff coming round. They can use it in their own time as and when they feel… *(Participant 10, Group 2).

*Choice and option are clearly the key to it, and that’s what virtually all of us would absolutely endorse* (Participant 3, Group 1).

Staff also spoke about the potential value of digital tools to provide people with a sense of ownership over their clinical data and the option to share this information with significant others in their lives, enhancing systemic ways of working with service users and their families. Staff were clear, however, that the choice about sharing data (or not) should be the service user’s decision:

*They can sort of choose who they use it with. It is sort of inclusive, as inclusive as you want it to be … you would be able to sit down and get the family involved; it could actually enhance the families’ understanding* (Participant 2, Group 1).

Specifically, staff felt service users might find digital healthcare solutions more acceptable than staff members themselves, as EIS service users are younger, more likely to own smartphones, and feel comfortable using them compared to people with more chronic mental health problems:

*A lot of our service users are, by virtue of their age, quite technically savvy *(Participant 45, Group 6).

Clinicians also felt that apps may be more acceptable for those in the early phase of psychosis. However, the time at which clinicians felt a digital health tool might be most helpful differed. For example, some clinicians suggested that an app might serve as a method to introduce service users to therapy [“It could be a good starting point. A good exposure exercise” (Participant 39, Group 5)], while others believed that an app may be more appropriate for people who had already experienced therapy and were further along in their recovery:

*Some of the people that we meet might not be at a place where they will be able to engage with [an app], whereas further down the line, it might be something that they are more able to benefit from* (Participant 46, Group 6).

Despite fears regarding the perceived loss of face-to-face contact associated with digital tools, some clinicians highlighted that these tools may be particularly acceptable for people who find communicating face-to-face challenging due to the opportunity to receive an intervention without the need to directly speak with another person:

*There were a few clients I thought it would work quite well with, like there’s a few who kind of spend a lot of time on a computer and don’t really go out much, and there’s some who don’t really want to interact with us … if they interact with an app, brilliant, that’s a head start* (Participant 25, Group 3).

Although the majority of clinicians felt they bring something, such as the therapeutic relationship, to therapy that digital platforms do not, some staff members recognized that the combined knowledge that can be distilled in something like an app might in fact be more effective than a single clinician delivering an intervention:

*It [a smartphone app] has got everybody’s knowledge and has great potential. “The whole is bigger than the sum of its parts” kind of thing. So it’s got information from all of us. So actually, it could be a huge resource in terms of knowledge and normalizing types of information* (Participant 3, Focus group 1).

*You are not dependent on one person’s training or understanding* (Participant 6, Focus group 1).

### Theme 3: Data Security, Safety, and Risk

The third theme is centered around concerns and considerations for the use of, access to, and response to data inputted into digital healthcare systems. When asked about governance, data protection, and security concerns, staff highlighted that potentially highly sensitive symptom data gathered by a digital device would need to be heavily protected and secured to ensure confidentiality and anonymity. Specifically, to prevent unauthorized access and individual identification, clinicians advised that digital tools need to be password-protected and that anonymous usernames should be used rather identifiable patient data:

*Perhaps if everything that is entered on there is anonymous so there is not the person’s real name anywhere, then that may be some reassurance* (Participant 2, Group 1).

Staff also described the need to specify where service user data would be stored and who would have access to these data in a clear, user-friendly, and accessible way:

*I think we just need to be really explicit about what the data’s for and where it’s going … you know when we say it’s in a database or in the team, what does that mean? Who’s going to have access to that?* (Participant 21, Group 3).

A common fear expressed by staff was the complexities of, and responsibility for, identifying and managing risk. Staff felt that such a “constant stream of information would be overwhelming … and impacts on whether we are able to respond” (Participant 17, Group 2), thus adding to already-stretched workloads. Additionally, some staff perceived that there would be a need to conduct robust risk assessments prior to “prescribing” a DHI to mitigate risks of an app triggering risk behaviors:

*If it does trigger an emotional feeling … something happens and we give them the app, then who’s responsible?…are we going to have to risk-assess to see if their mental health state is good enough? …[What if] something happens, and they have a bad turn and they say, “If I hadn’t gone in this app”? What is the impact on us?* (Participant 22, Group 3).

Integrating the near-constant stream of data into an electronic care record was also viewed with caution. Staff felt that real-time monitoring would require additional professional responsibility that they felt cautious about. While staff raised issues around access to, and responsibility over, risk disclosures using technology, they also felt that it was important to give service users the opportunity and space to freely express distressing experiences. Staff felt strongly that DHIs should include a diary function for service users to more freely express themselves, but with the caveat that it is made explicitly clear that clinicians will not be able to access, or be responsible for, this information:

*I would like an option for a space for people to express their distress … you would make it very clear to somebody that if they are going to write how they are feeling … it’s not going to be taken up by the therapist or anybody, but it is just there for the individual themselves to see it* (Participant 1, Group 1).

In addition to clearly stipulating the limits of clinician access to a prescribed digital tool, it was also suggested that the inclusion of emergency contact numbers may help mitigate the possibility of service users inputting risk information in the hope of getting support in order to place “the responsibility with the client for their safety” (Participant 4, Group 1).

### Theme 4: Relationships

Clinicians identified potentially positive and negative implications of digital technology on the staff–service user relationship. Specifically, clinicians expressed: i) doubts over the ability for a DHI to provide the relationship they considered vital for successful therapeutic care; ii) concerns that DHIs were dehumanizing and lacked the personal touch, genuineness, reciprocity, and warmth that another person can provide; and iii) the possibility that DHIs may reinforce avoidance of social situations. However, there was also the recognition amongst clinicians that, for some service users, digital healthcare tools may be preferable over face-to-face contact.

#### Absence of a Therapeutic Relationship

Many clinicians described the inherent need to build a therapeutic relationship with service users in order to deliver effective therapy. As digital technology potentially removes the need for human interaction, staff felt that the absence of a therapeutic relationship means that delivering therapy *via* a digital platform either was not possible or would be ineffective:

*I think if you are going to talk evidence-based, the biggest thing is every single intervention that has ever been tested is therapeutic relationship, so you are just removing the most effective part* (Participant 15, Group 2).

While this opinion was widespread, a few participants wondered whether clinicians, rather than service users themselves, place inflated importance on the impact of the therapeutic relationship, as Participant 4 in Group 1 says:

*But I guess therapists always hang on to the idea that the relationship is really important and part of the whole thing, but it’s not. I remember hearing this one idea that relationship is necessary but not sufficient. I heard somebody else say that actually, the relationship is sufficient on its own, but it’s not even necessary … So interventions can be delivered in any way really, and I’m starting to subscribe to the idea that potentially, it could just be a really effective thing. You don’t need a therapeutic relationship to do effective therapy.*

*I think that’s quite a paternalistic way of viewing it, really, to say we do the therapy to you. I think you just let people have a go at it. Sometimes what you find is the thing they identify as they go for treatment is not what you would identify, and that’s just as it should be* (Participant 45, Group 6).

#### Personal Communication and Connection

A common concern was that technology could seem mechanical and robotic due to the perceived inability to personalize and tailor responses to an individual. As such, staff were concerned that this would lead to reduced depth and quality of information. For example, DHIs cannot deliver more subtle or complex aspects of human interaction, and the ability to personalize information is compromised:

*[Technology] is very depersonalized … so much of our work and what lots of us think about is it’s always individualized, it’s always very personalized* (Participant 13, Group 2).

Staff also described how the warmth, support, and personal contact that just the presence of another person can have is important in recovery, which they felt is something that a DHI could “never offer”:

*I think if you asked clients what they valued most about their contact with the service, it would be the contact, it would be somebody coming around* (Participant 30, Group 4).

The power of nonverbal and para-communication when seeing people face-to-face allowed staff to identify a service user’s emotional responses, something they believed that a DHI was incapable of doing:

*All of those kinds of non-verbal cues and para-language is lost in electronic communication, and a lot of what we do relies on non-verbal cues and para-language. Saying that they’re fine and they’re still in their bedclothes that they’ve been wearing for three days … IT [information technology] would never tell me that* (Participant 31, Group 4).

Staff often spoke of therapy being one of the only opportunities that some service users had to socialize and connect with others. DHIs were viewed as potentially taking away the one chance an individual has to socialize:

*It could be the only socialization they get is with their therapist, and they’ve even taken that away or negated the need for that* (Participant 31, Group 4).

Additionally, staff spoke about the potential for apps to reinforce social avoidance, therefore affecting relationships in general in addition to therapeutic relationships with staff:

*There’s always an element if you want to be avoidant, of doing so, and I wouldn’t, I don’t know if there would be a risk that we kind of really reinforce that [avoidance] a bit here* (Participant 19, Group 2).

#### Digital Health Interventions Should be an Adjunct to, Rather Than Replace, Face-to Face Healthcare

Staff were concerned that service users might feel “fobbed-off,” neglected, or dismissed if they were prescribed an app. Technology was described by staff as a “de-humanizing” and “unhelpful” alternative to face-to-face care. Consequently, staff believed that service users would feel let down and therapeutic relationships would be adversely affected if users felt they were being given inferior care. Staff members suggested that one way of overcoming this would be for digital tools to be presented to people as an adjunct to, rather than replacement for, their healthcare:

*Some people might feel they are being fobbed off … but I think as long as you are able to fully explain what its purpose is and perhaps say this isn’t a replacement of a service, it is more an add-on, it would probably be seen as a bit more acceptable* (Participant 35, Group 5).

In fact, an almost universal opinion expressed by clinicians was that technology should not be used to replace traditional face-to-face care. Rather, technology should be used to augment existing support. Staff were able to identify concrete situations in which an app could be used to enhance the current care that they provide. For example, some staff noted that an app could be a useful and practical tool to deliver self-guided therapy materials between traditional face-to-face therapy sessions:

*It would be really useful for homework tasks and for supporting people to do things in between sessions rather than as a replacement for one-to-one sessions* (Participant 36, Focus Group 5).

Staff also valued the potential for DHIs to be used in conjunction with traditional mental health services to: i) monitor service users’ symptoms and experiences in order to identify early indicators of relapse, ii) aid diagnosis, and iii) provide clinicians with a more ecologically valid description of service users’ symptoms to inform subsequent care and facilitate shared decision-making:

*You’ve got something that can actually tell you that their mood has been getting better or worse, so it would … probably add more value, to the [therapy] in itself* (Participant 29, Group 4).

## Discussion

### Main Findings

This study examined clinicians’ views about adoption and use of digital tools within secondary care mental health service delivery. Four themes were evident within the data. First, a number of barriers to adoption and uptake of digital tools within services were expressed. Clinicians on the whole expressed the view that resources should be spent on more staff training rather than developing digital health products. In a time of austerity across Europe (24), it is not surprising that clinicians are concerned that digital tools are being used as a potentially cost-cutting exercise to limit resources being spent on staff training and/or staff employment. While a similar concern was expressed in another UK-based qualitative study with clinicians working in secondary care mental health services (1), it is a finding that is not limited to urban European-based community services. In a qualitative study of clinicians’ attitudes toward the use of online material in mental health service delivery in a rural Australian context, clinicians expressed concerns about the reallocation of investment into online resources at the expense of face-to-face service provision (9).

In the current study, fears were expressed around the potential for technology to usurp clinician-led care. Staff lacked confidence in using technology themselves and felt that this might impact on their own clinical judgment, although we found that younger clinicians reported more favorable views in embracing technology in their day-to-day practice than older clinicians. This may be because they themselves are more familiar with digital tools and use these more frequently in their own day-to-day life, perhaps experiencing some of the benefits digital tools afford. This supports findings in other studies that have also found that younger and more recently trained members of staff seem to readily accept integration of online approaches within their day-to-day practice (9). Furthermore, familiarity and ability of staff to use mobile technology and technology more generally has been shown to influence adoption of mobile health (mHealth) platforms across healthcare settings (25–29).

Doubts were expressed about technology being implemented in services at the organizational level. A number of staff commented on past failings of the NHS to implement technological solutions into electronic records and service workflows (15). This suggests that building confidence and trust in digital systems is an important issue to consider when implementing digital systems and pathways. In addition, the majority of staff felt that digital systems need to complement rather than hinder and complicate clinical workflows. Perceived usefulness and ease of use of digital tools were important factors found to influence mHealth adoption in a recent systematic review (30); this finding is reiterated in the current study and is important to consider when incentivizing staff to use integrated digital platforms.

Staff viewed digital technology as a generally acceptable, progressive, modern, and relevant approach to interacting with service users and implementing healthcare. These views reflect more general opinions expressed by clinicians across healthcare settings in other studies, where clinicians recognize that technology affords a new means of communicating with service users (30). Some staff recognized that they themselves might subscribe to more old-fashioned models of healthcare delivery and acknowledged that the younger digital native generation might feel more comfortable with using digital technology and might in fact see this as a preferred method of communication over face-to-face contact. A number of benefits were highlighted, including increased access to support in a manner unconstrained by time and location. Such on-demand access could potentially enhance the ecological validity of symptom/distress reporting. While the finding that mobile health (mHealth) systems can reach people anytime and anywhere has been echoed across the healthcare literature (30), it has not always been viewed as a benefit. For example, some studies of healthcare professional views about mHealth adoption in particular have shown that mobile technologies that embed real-time data into the clinician dashboard or into electronic workflows result in a sense of greater workload and disrupted workflow for staff, becoming a barrier to their adoption of mHealth systems [see (30) for a review].

As in previous studies (1), digital systems were also viewed as having the potential to improve social inclusivity, particularly for hard-to-reach groups. Although digital inclusion has improved in recent years (31), some people continue to remain digitally excluded. While digital solutions have the potential to bridge the healthcare gap and improve scalability of service, it is important that they promote inclusivity rather than further drive the social inequalities so evident among people with severe mental health problems. We support the assertion made by Robotham and colleagues (31) that a digital inclusion strategy is needed within health services to minimize rates of digitally excluded populations.

Clinicians perceived digital tools as potentially capable of reducing the stigma associated with a mental health problem, as apps are commonplace and socially accepted. Somewhat surprisingly, the issue around apps potentially normalizing the stigma associated with having a severe mental health problem has not been raised in other qualitative clinician studies in the field. In contrast, the role of mobile systems to support service user empowerment has been found across the healthcare literature (32, 33) and was reflected in the current study. A perceived benefit of digital systems was the potential to improve the sense of empowerment, control, and choice in healthcare pathways that traditional doctor/clinician and service-led care have historically not been able to provide. Data ownership, affording service users the choice in whether they wish to share digital data or not, was another perceived benefit of digital systems. Indeed, digital technology and the immediate and ubiquitous access to information, as well as intentional and unintentional digital authorship, have also changed how we engage with services and do indeed challenge the notion of data ownership, raising a number of ethical dilemmas that will require careful consideration when implementing digital systems into services (34).

A number of concerns around data security, safety, and risk were shared. Secure systems need to be in place and clearly articulated to reassure clinicians of safe and secure handling and storage of data. Security and risk concerns are commonly expressed as significant barriers to the adoption of digital tools and systems and are in fact concerns expressed by staff across a range of healthcare settings worldwide (35–37). For example, in a recent systematic review, Gagnon and colleagues (30) found that professionals are concerned about the security and confidentiality of data contained and transferred across technology platforms—such findings are echoed in our data. Concerns around data privacy and security were also expressed in another qualitative study of secondary mental health staff working with people with severe mental health problems (1), highlighting the importance of addressing such concerns to minimize barriers to adoption and implementation. In the wake of the General Data Protection Regulation (GDPR) data protection laws across the European Union (EU), the importance of protecting personal data is not only changing the landscape of regulated data protection law, but is also governing the standards with which services will be expected to control and manage personal data, which will undoubtedly raise concerns for digital workflows. On a related point, staff also felt that a clear procedure for managing risk that is potentially identified in real-time data workflows was needed. This echoes the findings of Berry and colleagues (1), which, similar to the current findings, showed that staff in secondary care mental health services raise concerns about their moral, legal, and professional obligation in assessing and managing risk in the context of digital monitoring systems. Guidance on how to respond to risk once identified was viewed as essential in making roles and responsibilities in reporting and responding to risk clear.

The final theme centered around the negative impact digital systems may have on the staff–service user relationship. Although some staff recognized that service users might in fact prefer digital methods of communication to face-to-face contact, by and large, staff were concerned that digital tools lack the nuance, warmth, and empathy a human can offer in easing distress. Concerns here and in another qualitative study of secondary clinician views of mHealth tools for service provision (1) were also expressed around the negative impact of digital tools on the therapeutic relationship, which was viewed as fundamental to improving outcomes. In a recent consensus document on the top 10 research priorities in a national study involving 600 mental health stakeholders in the UK, digital therapeutic alliance was voted into the top 10 research priorities for digital technology in mental healthcare (38). The introduction of technology into delivering therapy undoubtedly brings new challenges in service and therapy provision, particularly with regard to relationship building. There is some evidence that online therapy generates a similar therapeutic relationship to that observed and measured in face-to-face delivered therapy (39), and researchers are now starting to modify traditional measures of therapeutic alliance to capture alliance with online and mobile-based systems [see Ref. (40) for a review of digital mental health apps and therapeutic alliance]. More specifically, members of our group (41) adapted the Agnew Relationship Measure (ARM), a well-validated measure of therapeutic alliance in face-to-face therapy, and developed the Mobile Agnew Relationship Measure (mARM), which was found to have good face and content validity. A measure such as this can be used to advance our understanding of how therapeutic alliance influences outcomes in the context of DHIs. Nevertheless, blended approaches to digital system implementation in services may be more acceptable than stand-alone products, although the efficacy of this approach and whether it confers added benefit over and above stand-alone digital systems require further empirical investigation with both service users and services.

Relatedly, staff were concerned that incorporating digital systems into service delivery might negatively impact on the depth and quality of information shared and might in fact remove the only social contact a service user might have with the outside world. While the human element is indeed removed in the context of a digital system, there is considerable scope to supplement the loss in human connection by developing more precise algorithms and relapse detection methods, as is the promise with precision medicine (42).

Another concern expressed by participants was the potential for service users to feel dismissed and be given inferior care when prescribing digital products rather than face-to-face contact. Blended approaches that combine both digital systems and face-to-face contact were considered a preferable option to stand-alone digital tools being implemented. Our participants are not alone in this view. Clinicians interviewed in other qualitative studies expressed concerns that service users might feel neglected if they are referred to online or mHealth resources/packages and therefore advocate a preference for digital systems to be used as an adjunct rather than an alternative to face-to-face therapy and support (1, 9).

### Strengths and Limitations

There were a number of strengths in the current study. We had heterogeneity within groups (e.g., staff of various ages/disciplines) yet homogeneity between groups; it is likely that a variety of views and attitudes were captured. We also had full representation from an entire local early intervention team in three of the six focus groups conducted in this study, thereby minimizing selection bias. On the same note, we acknowledge that for the remaining three focus groups, staff who held a particularly strong view about DHIs may have self-selected to take part in the study, thereby influencing the representativeness of the sample. We adopted a stringent approach to data analysis involving member checking with the majority of our focus groups and triangulation of data. The close working relationships observed within focus groups might have enabled participants to feel more comfortable speaking openly and honestly about their views.

There were also some limitations. We used a purposive sampling approach. Although conducted across three large NHS Trusts, findings reflect views expressed by staff working in the Northwest of England; staff views/attitudes about digital healthcare may be different elsewhere. Three of the six focus groups were from the same service; views from this team particularly influenced the data, and so findings might reflect this particular team’s views rather than clinicians’ views more broadly. A strength of the focus group design is that it allows people to generate ideas through discussions with each other. However, an associated limitation is that data generated are dependent on the individuals within each group; individual perspectives impede the expression of a variety of views. To minimize the interdependency of participants, group dynamics were managed by the interviewers such that all members of the group were encouraged to express their views. Finally, this was a qualitative study nested within a broader research program on the development and testing of a theory-informed smartphone app for early psychosis, *Actissist* (4). Staff were aware that this project involved developing a DHI for early psychosis, and so views expressed may be filtered.

### Implications

In our study, staff recognized a number of potential benefits to embracing digital tools and systems when working with people with severe mental health problems. In particular, staff expressed the view that apps are easily accessible and unconstrained by time and location, affording the capacity to deliver support and intervention to scale while empowering service users in their healthcare. The on-demand access inherently available within a DHI can provide an ecologically valid assessment of symptoms. Furthermore, staff acknowledged that digital tools could engage harder-to-reach groups of people who struggle to access traditional services.

In line with our findings, we provide a list of practical recommendations in **Table 3** for services and organizations to consider when implementing digital systems within routine mental health service delivery. However, there are some recommendations we wish to highlight here as particularly important. Firstly, our data suggest that staff fears around the role of technology in service delivery need to be openly discussed when teams are considering incorporating digital tools in services. That is, normalizing concerns, problem-solving solutions/safeguards, and providing evidence to alleviate concerns in a supportive, nonconfrontational manner are important for successful implementation. Staff training in using digital tools is just as important as training service users, not just in practical terms but also by way of increasing clinician confidence and familiarity with digital technologies. Importantly, digital systems need to adhere to strict data management procedures, ensuring that systems are secure and safe. Services need to describe in clear and simple terms how digital data will be stored and who will have access to these data. In the UK at least, mental health professionals are using digital systems routinely as part of note keeping in service users’ electronic health records. Shortly, healthcare professionals in the community will be accessing these records on their mobile devices. Organizations need to support cultural and attitudinal change using digital technologies in mental healthcare if successful implementation of the government’s Paperless 2020 and Digital NHS strategies is to take effect. There is a significant amount of work needed for NHS services (and services internationally) to move and align their information technology systems into the modern digital world and to equip staff with the training required. To understand the benefits of the process and for services to feel able to adopt, implement, and deliver a digital NHS, staff need to be fully engaged in the process. A culture shift is required to embrace technology into routine service delivery in order to fully implement digital workflows and systems into policies and commissioning frameworks. In line with the recent consensus statement about appropriate standards, principles, and practices in research and evaluation of digital tools and systems (44), and as recommended by Bucci and colleagues (45), rigorous evaluation of DHIs is also another critical step for real-world integration. Findings from the current study go some way in telling us how the rollout of digital tools will affect clinical practice in specialist mental services in the public health service model of healthcare.

**Table 3:** Recommendations arising from these findings.

Recommendation	
**Recommendations when considering impact of digital tools on staff and services**	Staff fears around the role of technology in service delivery need to be discussed up front and addressed when considering incorporating digital tools in services. E.g., normalizing concerns, problem-solving solutions/safeguards, and providing evidence to alleviate concerns in a supportive, nonconfrontational manner are important.
	Training staff in using digital tools is equally as important as training service users, not just in practical terms but also by way of increasing clinician confidence and familiarity with digital technologies.
	Organizational support with a clear plan for implementing technological innovations is required, with targets in the implementation plan that are assessed and measured.
	Implementing of digital systems needs to be simple and uncomplicated and improve clinical workflows rather than hinder and increase clinical workflows.
**Recommendations when considering impact of digital tools on Service users**	Although ownership rates of mobile phones in psychosis are comparable to the general population (43), for those who do not have access to smartphones, services might consider loaning phones to negate the digital divide.
	Digital products need to be made available in multiple languages as well as in different mediums (e.g., audio and video) to not further facilitate social exclusion in an already-marginalized group.
	Digital systems should use schedule and prompts to engage services users with the products and consider using social media platforms to facilitate connection and communication with others.
	Emphasize to services/staff the positive aspects of digital systems (e.g., increased access to support; improved social inclusivity; more ecologically valid reporting of symptoms/distress; reduced stigma; digital technology is often more user-friendly for “digital natives” and the preferred method of communication for this group).
**Data security, safety, and risk**	Digital systems need to adhere to strict data management procedures, ensuring that systems are secure and safe. Services need to describe in clear and simple terms how digital data will be stored and who will have access to these data.
	A clear procedure for managing risk, especially in the context of real-time data workflow streams, is needed.
	At a minimum, simple features like emergency contacts built into digital systems may help both staff and service users feel supported when clinicians are not able to respond to immediate signs of risk.
**Impact of digital systems on relationships**	Blended approaches to implementing digital systems into services may be more acceptable to clinicians than stand-alone digital products.

## Data Availability Statement

The datasets for this manuscript are not publicly available because consent is not obtained. Requests to access the datasets should be directed to sandra.bucci@manchester.ac.uk.

## Ethics Statement

This study was carried out in accordance with ethical approval from the National Research Ethics Committee West Midlands—South Birmingham (14/WM/0118) and with written informed consent from all subjects. All subjects gave written informed consent in accordance with the Declaration of Helsinki. This study was carried out in accordance with ethical approval from the National Research Ethics Committee West Midlands—South Birmingham (14/WM/0118). The study was prospectively registered (ISRCTN34966555) and received.

## Author Contributions

SB conceived of the study and led on study design, analysis, and writing the manuscript. DE contributed to study design, oversaw data analysis and development of the framework, and approved the final manuscript. RM and NB contributed to data analysis and interpretation of results and approved the final manuscript. KB and GH independently reviewed study themes, contributed to study design and interpretation of results, and approved the final manuscript. SL contributed to editing the manuscript and approved the final version.

## Funding

The work was supported by the Medical Research Council Developmental Pathway Funding Scheme (grant number MR/ L005301/1). The work was supported by the University of Manchester, Greater Manchester Mental Health NHS Foundation Trust, and the Clinical Research Network.

## Conflict of Interest Statement

SB and SL are directors of a not-for-profit community interest company spun out of the University of Manchester designed to make digital health apps commercially available in the UK National Health Service. The remaining authors declare that the research was conducted in the absence of any commercial or financial relationships that could be construed as a potential conflict of interest.

The reviewer JF declared a past coauthorship with one of the authors SB and SL.
